# Use of Fibrin Glue in the Treatment of Persistent Pneumothorax in Premature Infants at the Limit of Viability: Ethical Issues and Two and A Half Years Follow-Up

**DOI:** 10.34763/jmotherandchild.20232701.d-23-00061

**Published:** 2023-11-22

**Authors:** Magdalena Rutkowska, Martyna Woynarowska, Iwona Terczyńska, Małgorzata Seroczyńska, Dariusz Mydlak, Jarosław Mądzik, Ewa Nowakowska, Katarzyna Niepokój, Sławomir Szczepaniak, Krystyna Polak

**Affiliations:** Department of Epidemiology and Biostatistics, Institute of Mother and Child, Warsaw, Poland; Neonatal Intensive Care Unit, Bielanski Hospital in Warsaw, Poland; Neonatal Outpatient Clinic, Institute of Mother and Child, Warsaw, Poland; Clinic of Surgery of Children and Adolescent, Institute of Mother and Child, Warsaw, Poland; Department of Diagnostic Imaging, Institute of Mother and Child, Warsaw, Poland; Otolaryngology Clinic, Institute of Mother and Child, Warsaw, Poland; Department of Medical Genetics, Institute of Mother and Child, Warsaw, Poland; The Catholic Academy in Warsaw, Collegium Joanneum, Warsaw, Poland

**Keywords:** premature infant at the limit of viability, fibrin glue, pneumothorax, ethics

## Abstract

**Introduction:**

Due to the extreme immaturity of many internal organs, including lungs, infants at the limit of viability are more predisposed to a pneumothorax (PTX). In some cases, PTX becomes persistent. Previously, only a few attempts of PTX treatment with fibrin glue were reported. However, its impact on further lung development is unknown.

**Case report:**

We present a case of an extremely preterm infant with persistent PTX who was successfully treated with fibrin glue. In addition, we present a two-and-a-half-year corrected age follow-up focusing on respiratory problems, motor development and sensory organs. Furthermore, we touch upon the related ethical issues.

**Conclusions:**

Fibrin glue should be used to treat persistent PTX even in an extremely preterm infant. No adverse effects were observed. At the two-and-a-half-year corrected age follow-up, despite severe bronchopulmonary dysplasia development, no serious pulmonary problems were observed. However, the child's development is uncertain. This situation raises important ethical issues concerning saving the lives of infants at the limit of viability.

## Introduction

In a group of ‘foetal’ neonates (<26 weeks of gestation), numerous questions are still asked: Which week of gestation should the mother be given prenatal steroids? [1] Where is the limit to undertake resuscitation in the delivery room or take the newborn into palliative care? What should be taken into account when making these decisions? What is the role of parents? When does treatment become futile during hospitalisation in the Neonatal Intensive Care Unit (NICU)? These are just some of the dilemmas doctors face when undertaking life-saving measures for newborns weighing <750 g [2].

Due to significant lung immaturity, these newborns are at greater risk of developing respiratory distress syndrome (RDS) and bronchopulmonary dysplasia (BPD) at birth. While in early life, they have an increased risk of pneumothorax (PTX), which is a severe and sometimes even life-threatening complication. The increasing use of prenatal steroids and exogenous surfactant intake after birth have resulted in a significant PTX reduction in these children, which is now approximately 1–2% [3]. The classic treatment for symptomatic PTX is a suction drain placement; neonates require endotracheal intubation and mechanical ventilation. If the trocar is not removed by day 7, such a PTX is defined as persistent and favours severe BPD development [4]. In extremely immature neonates, the use of pleurectomy or selective intubation of the contralateral bronchus is not possible, and therefore, fibrin glue is attempted in exceptional cases. To date, 12 cases of extremely immature neonates receiving this type of treatment have been described. The glue was first administered in 2012 [5], and the youngest patient described was born at 24 weeks of gestation [6]. In the published articles, alongside the description of respiratory problems, only data on the survival rates and the neonatal status at hospital discharge are presented. However, there is no information on the subsequent respiratory problems and development of these children.

We present a case report of a neonate born at the limit of viability who was treated with fibrin glue due to persistent PTX and whose development was assessed after two and a half years of life. At the same time, we wish to draw attention to the ethical problems associated with treating these extremely immature neonates.

## Case report

### a. From birth to discharge home with particular attention paid to pulmonary problems

A female neonate was born by spontaneous labour at 22 weeks of gestation + 6/7 days. At 20 weeks of gestation, the mother was diagnosed with Premature Rupture of Membrane (PROM), followed by anhydramnios. She received antibiotic therapy due to C-reactive protein (CRP) rise and did not receive prenatal steroid therapy due to extreme prematurity (< 23 weeks of gestation) and an estimated foetal body weight of 480 g. Before delivery, we discussed with the parents the possibility of providing palliative care for the baby in the delivery room according to the Polish recommendations [7], as the only factors improving the prognosis were singleton pregnancy and female gender.

At birth, the baby had an Apgar score of 1 (heart rate 90–110 bpm) and a bodyweight of 630 g (90th percentile). Resuscitation efforts were undertaken: the baby was placed in a plastic bag; Neo–Puff with FiO_2_ of 1.0 was applied. At 3 minutes of life, the baby was intubated. Next, the patient received surfactant (200 mg/kg of body weight Curosurf, Chiesi Farmacetuici Spa, Parma, Italy) endotracheally and mechanical ventilation in SIMV mode with the following parameters: PIP 28, PEEP 5, frequency 60 per minute, Ti 0.35, FiO_2_ 1.0. Saturation was maintained between 90–95%.

**Figure 1.**
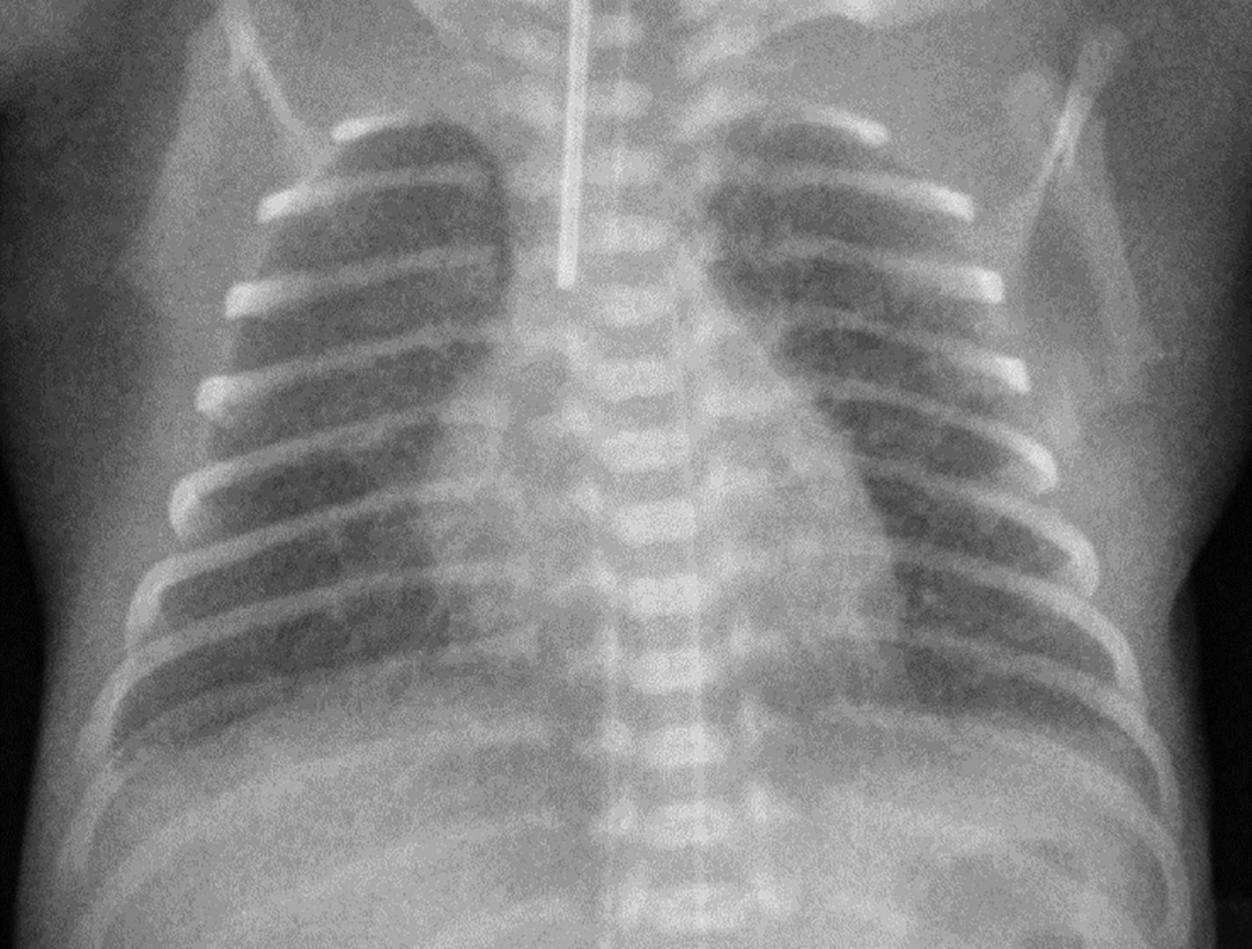
Anteroposterior radiograph shows an intubated 23-week premature infant at the 5^th^ hour of life with respiratory distress syndrome (RDS) showing pulmonary hypoventilation and granular densities bilaterally. Note the incorrect position of the tip of the arterial catheter.

**Figure 2.**
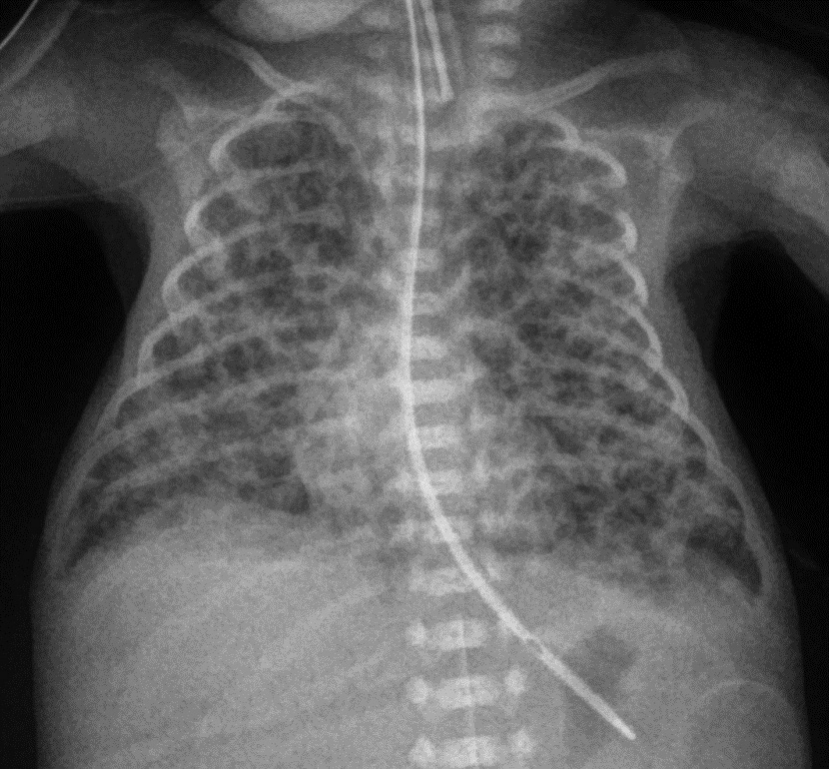
AP chest radiograph 8 days later shows bubbly lucencies in the interstitium throughout both lungs with hazy background opacities. These findings are typical of diffuse bilateral PIE (pulmonary interstitial emphysema) in the setting of RDS.

**Figure 3.**
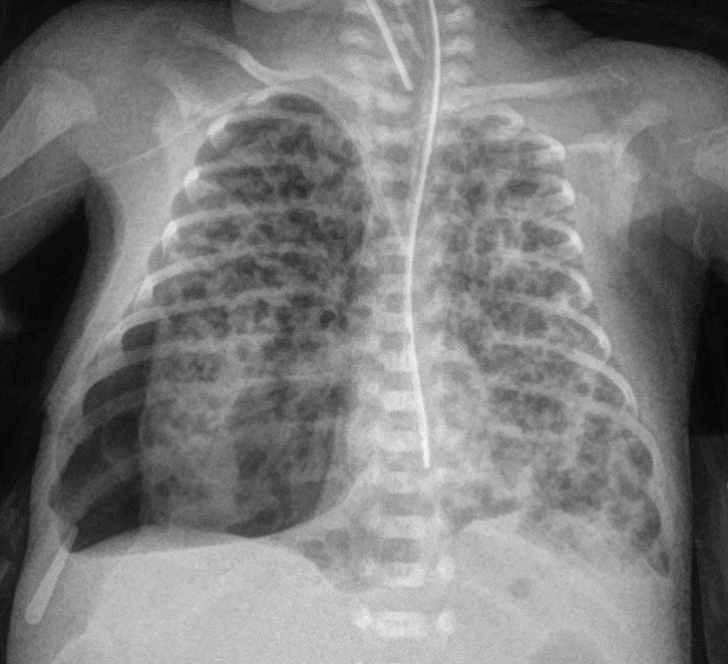
Supine chest radiograph on the 14^th^ day of life shows a lucent right hemithorax, depression of the right diaphragm and leftward mediastinal shift, consistent with a right mild tension pneumothorax.

**Figure 4.**
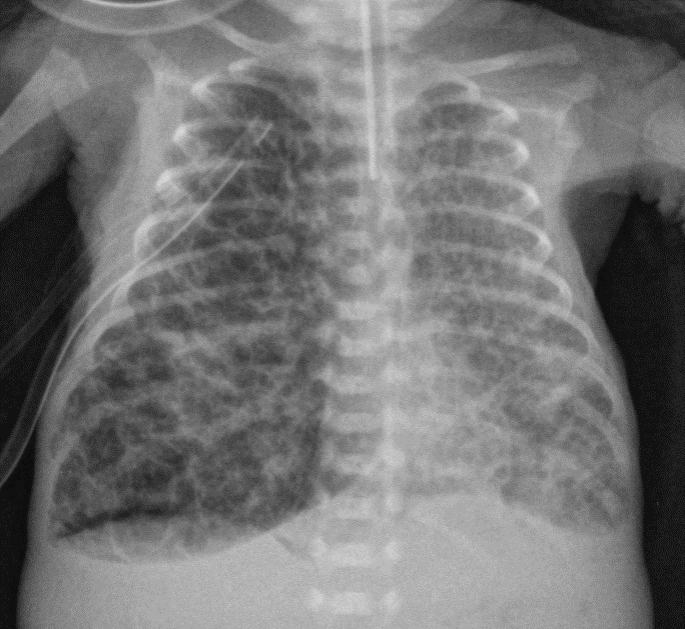
Supine chest radiograph at 25 days of age with chest tubes placement shows persistent extensive bubbly lucencies in both lung (PIE) and resolution of the most of right pneumothorax (slight shift of the heart leftward pressing the left lung and the depression of the right hemidiaphragm is still visible).

At 5 hours of life, the chest X-ray showed features of grade 2 RDS (Figure 1). The PC/AC + VG (Pressure-control/Assist control + Volume Guarantee) mechanical ventilation with variable parameters was carried out. From the 5^th^ day of life, chest radiographs presented increasing interstitial emphysema features with parenchymal opacities (Figure 2). The patient's ventilation was changed to high-frequency oscillatory ventilation (HFOV) mode with no improvement. On the 14^th^ day of life, a right-sided PTX was diagnosed (Figure 3). The ventilation parameters were modified with a periodical repeat of HFOV mode. In addition, the child required constant oxygen to maintain the FiO_2_ of 1.0. The PTX was decompressed several times without achieving complete decompression on chest X-rays. Therefore, the trocar could not be removed (Figure 4). From time to time, the child required the supply of catecholamines due to accompanying hypotension.

**Figure 5.**
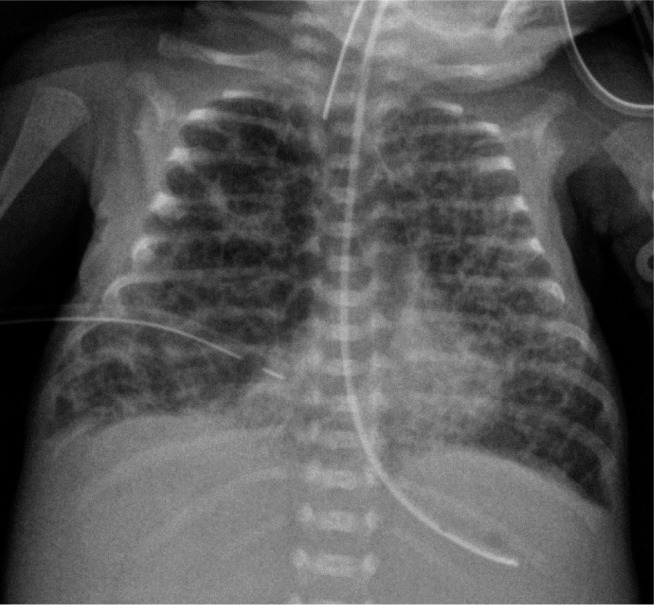
AP chest radiograph 1 hour after fibrin glue application via drain shows resolution of right pneumothorax. Persistent diffuse bilateral PIE. The left lung is better aerated than previously. A fibrin glue clot at the tip of the drain is observed.

**Figure 6.**
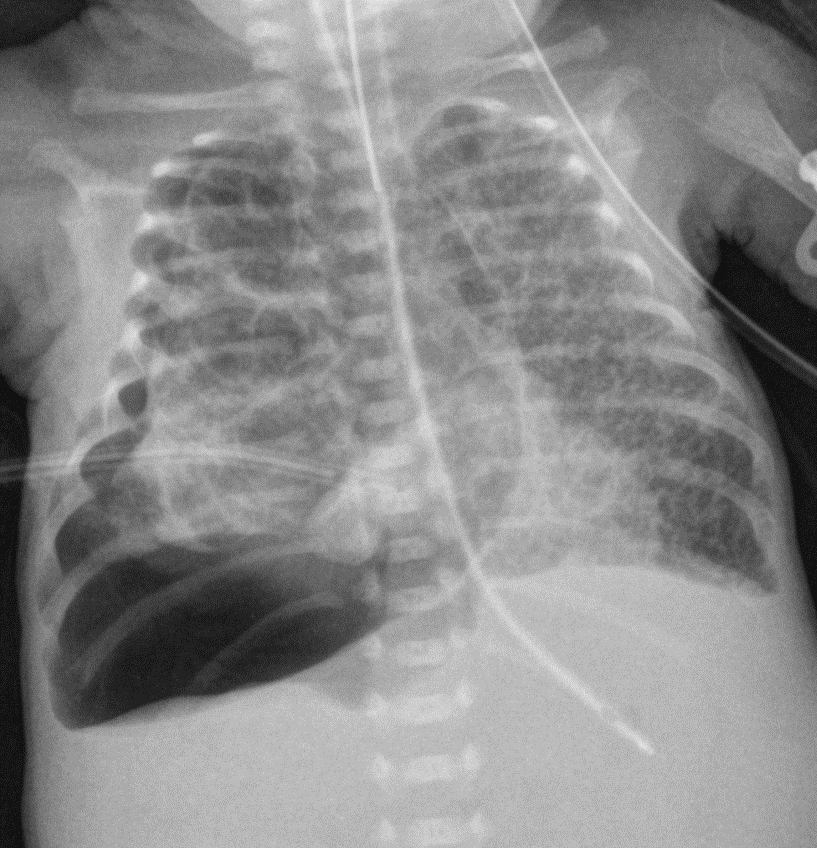
AP chest radiograph 2 days after fibrin glue application shows recurrence of tension right pneumothorax.

**Figure 7.**
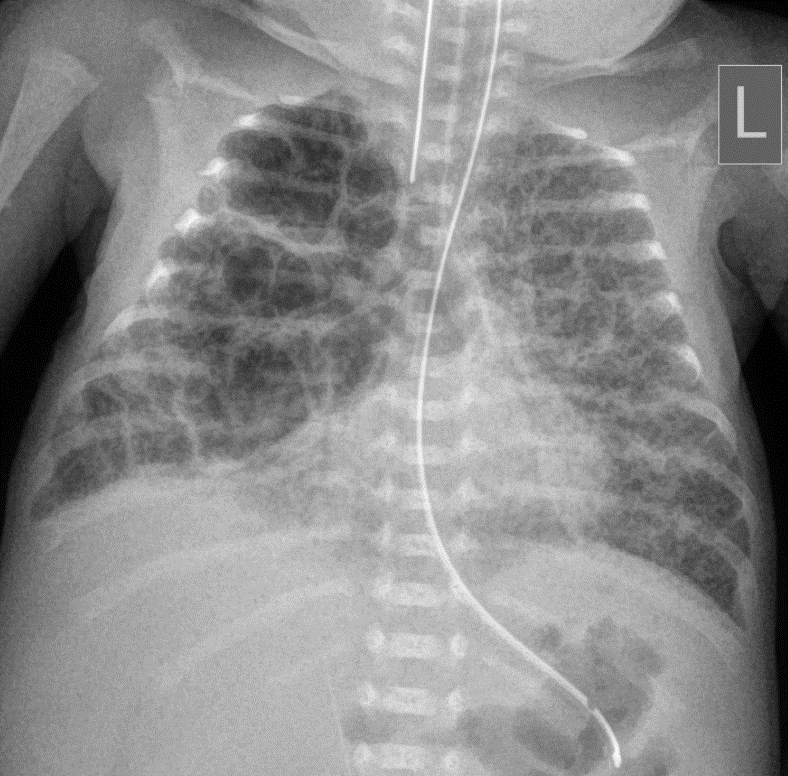
AP chest radiograph on the 46^th^ day of life (8 days after fibrin glue application) shows resolution of right pneumothorax (drain was removed). Marked hyperinflation with coarse interstitial markings and cystic changes in the right lung; signs of PIE in the left lung.

**Figure 8.**
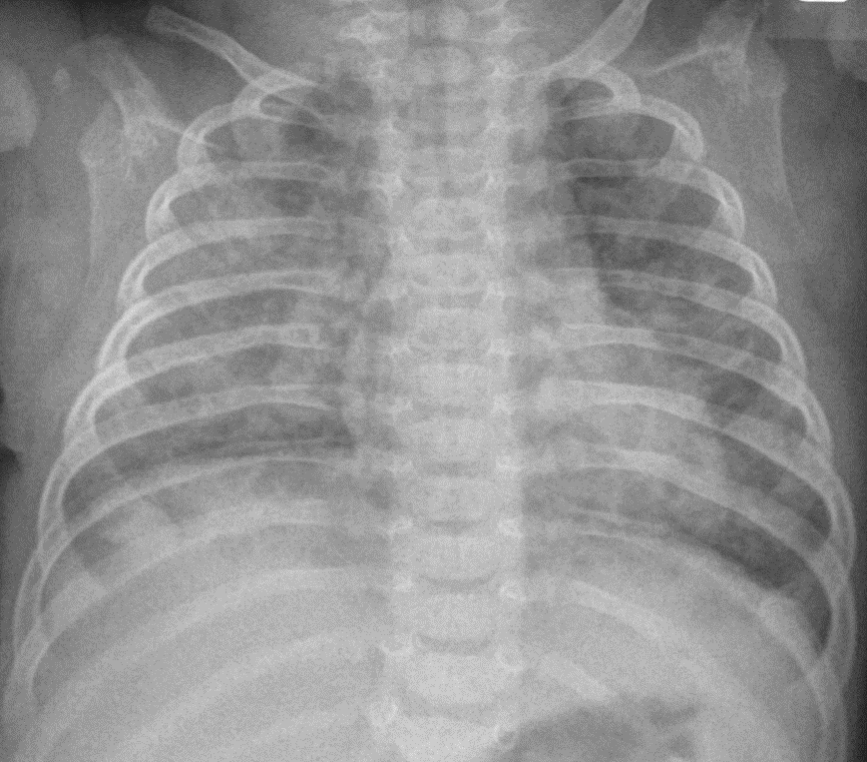
AP chest radiograph at the age of 7 months, before discharge home from the hospital. Generalized diffuse, hazy opacities and coarse interstitial markings with a small degree of hyperinflation – signs of bronchopulmonary dysplasia (BPD). Fibrin glue clot disappeared.

Based on the world literature data, on the 38^th^ day of life and after obtaining the parents’ written consent and approval from the local Bioethics Committee at the Institute of Mother and Child, Warsaw, Poland (opinion number 8/2021), a treatment with intrapleural injection of fibrin glue (Tisseel LYO) was attempted. Fibrin glue is a two-component material consisting of concentrated human fibrinogen and thrombin imitating the body's coagulation process [8]. The homologous fibrin glue (Tisseel Lyo, Baxter) was prepared in a pre-filled double-chamber syringe, which contained sealer protein solution (with synthetic aprotinin) in one chamber and thrombin solution (with calcium chloride) in the other chamber, resulting in 4 ml of ready-to-use product. The administration procedure was similar to previous reports [6]. During a 5-minute administration of 4 ml of glue through a catheter placed in the right pleura trocar, a desaturation episode down to 83% was observed; however, the heart rate was maintained above 120 bpm. Next, the trocar was clamped for 10 minutes. A chest X-ray performed 1 hour after drug administration (Figure 5) showed the complete resolution of PTX; however, the FIO_2_ level was not decreased. Unfortunately, on the second day after the procedure, the PTX reappeared (Figure 6), but the child's condition remained stable. Regarding that, the decision about fibrin glue readministration was not taken. Finally, the PTX ultimately resolved on the 46^th^ day of life, that is, day 8 after treatment administration (Figure 7). On the 62^nd^ day of life, the child was extubated. Up to the 92^nd^ day of life, the patient initially received non-invasive ventilation with a maximal FiO_2_ of 0.7; next, she received bi-nasal CPAP, nCPAP (Continuous Positive Airway Pressure) with FiO_2_ 0.6. From day 92^nd^ (at 36 weeks post-conceptional age), breathing was supported with Optiflow with FiO_2_ of 0.6 down-titrated to 0.35 under saturation monitoring. Subsequently, ambient oxygen therapy was used. A chest X-ray at discharge (Figure 8) showed typical BPD changes with a complete disappearance of fibrin glue clot. During hospitalisation, the child received one course of hydrocortisone (8 days), one course of Dexaven [*dexamethasone*] (6 days) and one course of Celestone [*betamethasone*] (7 days). This treatment was followed by Berodual [*fenoterol* + *ipratropium bromide*] and Pulmicort [*budesonide*] inhalations. Caffeine citrate (Cooper, France) was used for apnoea prevention. Periodically, the child was also receiving diuretics (Spironol [*spironolactone*], Gedeon Richter, Poland). At 36 weeks of post-conceptional age, a severe form of bronchopulmonary dysplasia was diagnosed. The patient received five doses of palivizumab (Synagis, AbbVie) to prevent Respiratory Syncytial (RS) virus infection. During hospitalisation, the patient experienced two systemic infection episodes with a methicillin-resistant coagulase-negative *Staphylococcus* (MRCNS) (total 21 days of treatment with vancomycin). The patient was diagnosed with retinopathy of prematurity (ROP), which was treated with two administrations of Lucentis [*ranibizumab*] (Novartis, Europharm) and laser photocoagulation resulting in the cessation of disease symptoms. During physiological sleep, the child underwent a hearing evaluation using DPOAE and ABR screening tests. No response to the DPOAE test was observed. In the ABR test, the hearing threshold was assessed based on the V wave – 80 dB bilaterally. No cochleopalpebral reflex was noted. Regarding the screening tests results, the diagnosis of profound bilateral sensorineural hearing loss was established. We excluded one of the most common genetic causes of non-syndromic hearing loss – mutations in the GJB2 gene.

**Figure 9.**
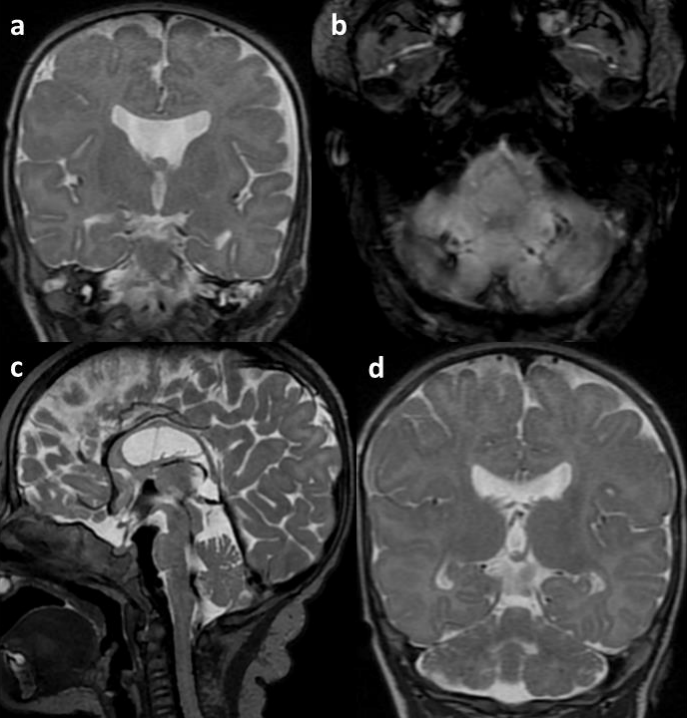
MRI of the brain at 14 weeks of corrected age. Lack of the septum pellucidum (a). Posthaemorrhagic haemosiderin deposits – black dots – in both cerebellar hemispheres on SWI sequence (b). Abnormal proportions of the cerebrum and cerebellum with decreased cerebellar volume on sagittal (c) and coronal (d) T2-weighted images.

Brain MRI performed at 14 weeks corrected age showed the following abnormalities: post-haemorrhagic haemosiderin deposits in both cerebellar hemispheres and vermis and decreased cerebellar volume. MRI scans also demonstrated absence of the septum pellucidum and thinning of the corpus callosum (Figure 9). The girl was discharged home after 250 days of hospitalisation on passive oxygen therapy with the FiO_2_ of 0.3.

### b. Evaluation at two-and-a-half-years corrected age

**Figure 10.**
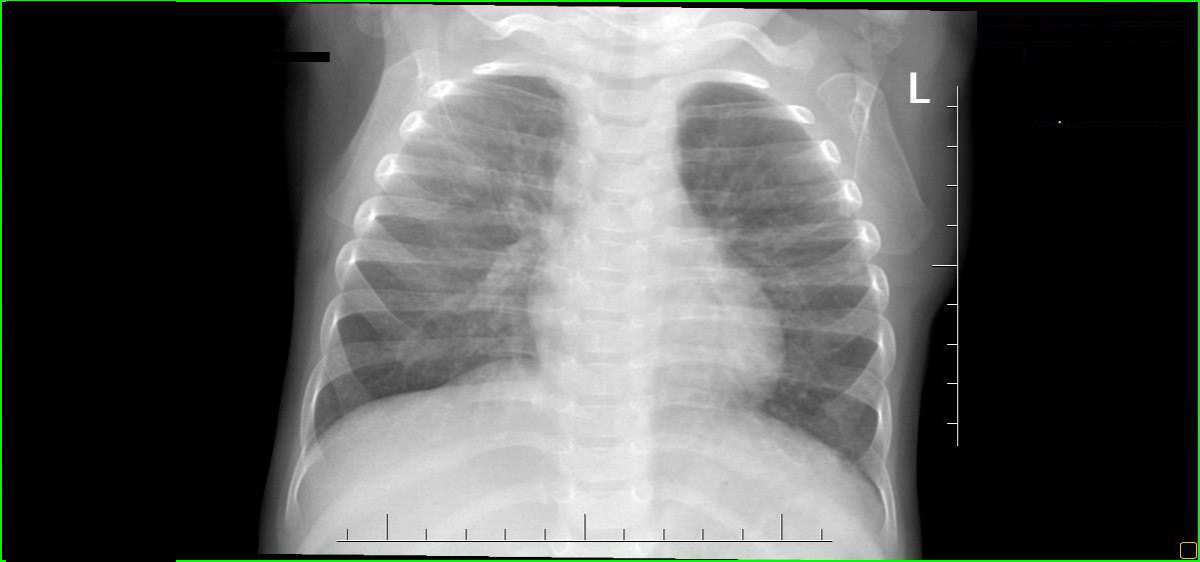
At 11 months of age, AP chest radiograph shows an improvement of lungs condition, which are mildly hyperinflated with only sparse linear interstitial markings bilaterally.

Between the discharge from hospital (19 weeks corrected age) and the end of 1 year corrected age, the child experienced only two catarrhal infections, including one episode of increased body temperature and desaturations requiring oxygen supply. For this reason, the child was hospitalised for 1 day. The chest X-ray performed (Figure 10) did not show any abnormalities. The respiratory function was satisfactory with normal vesicular breath sounds on auscultation without obstruction features. Require oxygen supply was used periodically up to 6 months. The patient was slowly gaining weight (12^th^ percentile) and, head circumference (around 10^th^ percentile), and no oropharyngeal abnormalities were observed. There was a hospitalisation at 2 years of age due to an incident of febrile convulsions in the course of a viral infection with a normal EEG.

At the age of one and a half, the muscle tone in the lower limbs’ had a slight stiffness, flaccid axial muscle tone, poor motor skills and markedly abnormal movement patterns were found on neurological examination. Currently, there is delay in gait development: the child crawls, gets up with equipment, walks with help, and has poor hand–eye coordination. There is delayed development of active speech, and the child understands commands and executes them with great difficulty in focusing attention.

Conclusion: There is psychomotor developmental delay with observation towards mixed cerebral palsy. The screening for autism spectrum disorders was negative. The absence of the septum pellucidum and cerebellar hypoplasia on MRI scans are unfavourable prognostic factors regardless of the burden of extreme prematurity and its complications, including distant ones. The ophthalmologic examination carried out at the age of one showed the function of the normal eye for that age.

At 8 months of corrected age, the child was fitted with hearing aids due to bilateral hearing loss. The child wears hearing aids on both sides during diagnostics for possible implantation.

## Discussion

Our case, in which fibrin glue was used in an extremely immature neonate, demonstrates that even when significant interstitial emphysema was found in a chest X-ray and the oxygen supply to maintain FiO_2_ of 1.0 and to provide proper saturation, such an attempt was worthwhile. This is also supported by the fact that the drug tolerance during administration was good, and the treatment efficacy, both clinical and radiological (chest X-ray at discharge and later), was satisfactory. Simultaneously, we did not observe any of the previously described side effects, such as bradycardia, hypercalcaemia (serum Ca level before fibrin glue administration was 2.35 mmol/L; a few days after administration 2.46–2.48 mmol/L) or skin necrosis [9]. It seems that the hypercalcaemia, as suggested by the authors, was related to the high calcium concentration in the previously used agent. We used Tisseel Lyo, which contains only 80 μmol of calcium chloride in 4 ml.

Despite the resolution of PTX after treatment with fibrin glue, we could not avoid complications in the form of a severe BPD and almost a year of oxygen supply (up to 260 days of age, periodically only during the night period). Could this be due to the too late administration, that is, on the 24^th^ day after PTX development? In the previously reported cases [5,6,9], the mean time to fibrin glue administration was shorter (range 8–16 days), but all the children still developed BPD, and two died. Therefore, it seems that, in our case, it was of little importance. On the other hand, significant interstitial emphysema, observed from the 5^th^ day of life, most probably resulting from lung hypoplasia associated with prolonged PROM and anhydramnios diagnosed a few days before delivery, have been the reason for PTX. However, it is comforting that, during the first year of life (corrected age), the child had only two catarrhal infections; stayed once in the observation ward for this reason (1 day), and the consulting pulmonologist did not find any auscultatory changes in the lungs at the age of 1 year; and the chest X-ray, except the hyperinflation of the lungs, was normal. Indeed, the administration of a full course (five doses) of palivizumab in RS virus infection prophylaxis was important.

How do we assess the global development of our patient? Certainly, the presence of bleeding into both cerebellar hemispheres as well as its resulting atrophy observed on brain MRI scans is worrying. The finding of delayed motor development, especially gross motor skills, probably results from this complication. We are also concerned about a predisposition of developing autism, which has been highly emphasised in the literature in recent years [10].

It is very reassuring to see the visual examination and a good response to the ROP treatment applied to attain an age-appropriate visual function in evaluating the sensory organs. However, the severe degree of hearing loss is worrying, although hearing aids used from the age of 8 months improved the situation, and the girl started pronouncing syllables a few weeks after their application.

Finally, it is impossible not to ask the ethical questions. The first ethical problem we encounter in this case report is the rightness to save a newborn born at 22 weeks of gestation. There is a lively debate in the literature on this issue [11]. Until a few years ago, most neonatologists believed that this age of neonatal survival – arbitrarily adopted by the WHO for statistical purposes – was of a purely theoretical nature, which made it possible to distinguish miscarriage from birth. The vast majority of doctors accepted the 24^th^ week of pregnancy as the newborn's age of survival, and the 23^rd^ week was considered as the grey zone. Today, some neonatologists declare their readiness to save newborns born even at 22 weeks of gestation [1,2,12]. The Polish medical recommendations considering ethical issues in the care of the mother and the extremely immature neonate say that a child born at 22 weeks of gestation should theoretically receive palliative care after birth. However, the parents’ wishes are also taken into account as well as factors improving the child's prognosis [7]. Therefore, bearing in mind the PROM found at 20 weeks of gestation with associated anhydramnios and the expected birth weight of 480 g, a prenatal interview with the parents was conducted, presenting them the possible events in the delivery room, including the possible death of the baby. After birth, however, it turned out that the weight was much higher (620 g), which could be due to difficulties in its calculation (anhydramnios). Two more factors were found to improve prenatal prognosis (in addition to singleton pregnancy and female sex observed in the prenatal period): higher birth weight relative to the predicted value and heart rate >100 bpm. Therefore, the decision to resuscitate the child was taken. Unfortunately, our case cannot confirm the thesis according to which the technological progress in neonatology in recent years guarantees children's survival at 22 weeks of gestation. So far, in the literature we find single cases of normal development of children born at this gestational age, and their evaluation includes only a few years [10,11]. In most cases, the development of these children significantly deviates from the norm. Our case also confirms this observation.

The second problem was the experimental administration of fibrin glue for treating PTX. In the literature, the youngest child undergoing this treatment was born at 24 weeks of gestation [6]. In our case, the classical treatment by trocar placement was ineffective. Only applying fibrin glue finally led to the PTX closure, improved the baby's condition, and enabled the lungs’ natural development supported by the previous administration of surfactant in the delivery room. It is worth noting here the medical and ethical difference between treating and supporting the natural process of maturation of individual organs of the extremely immature neonate.

The third ethical problem is to what extent the intensive treatment of an extremely immature newborn is justified, knowing that it may cause side effects (e.g. administration of catecholamines, sedation drugs, antibiotic therapy). To what extent can we accept a medicine (*pharmakon*) that becomes a poison (*pharmakon*) for the extremely immature neonate? Undoubtedly, treatment must be proportionate to the condition of the patient and the immaturity of their organs. In our case, we cannot state as to whether the child's deafness later on was due to the long-term antibiotic therapy (vancomycin) or is due to extreme prematurity. We excluded one of the most common genetic causes of non-syndromic hearing loss – mutations in the GJB2 gene.

## Conclusions

Fibrin glue should be used to treat persistent PTX even in an extremely preterm infant with significant lung injury. No adverse effects were observed during its administration. At the two-and-a-half-years of life follow-up, despite severe BPD development, the patient does not have any serious pulmonary problems. However, the child's motor development, assessed at the age of one, is uncertain. This situation raises important ethical issues concerning saving the lives of infants at the limit of viability.
